# Effects of dietary supplementation with *Bacillus velezensis* on the growth performance, body composition, antioxidant, immune-related gene expression, and histology of Pacific white shrimp, *Litopenaeus vannamei*

**DOI:** 10.1186/s12917-024-04207-4

**Published:** 2024-08-16

**Authors:** Arwa E. M. Abdelsamad, Rashad E. M. Said, Mona Assas, Alkhateib Y. Gaafar, Awatef H. Hamouda, Aldoushy Mahdy

**Affiliations:** 1 Zoology Department, Faulty of Science, Al-Azhar University, Assiut Branch, Assiut, 71524 Egypt; 2 Fish Processing and Biotechnology Department, Faculty of Aquatic and Fisheries Sciences, Kafrelsheikh University, Kafrelsheikh, 33516 Egypt; 3 Hydrobiology Department, Veterinary Research Division, National Research Centre, El Buhouth St, Dokki, Cairo, 12311 Egypt; 4 Fish Health and Diseases Department, Faculty of Fish and Fisheries Technology, Aswan University, Aswan, 81528 Egypt

**Keywords:** *Bacillus velezensis*, Body composition, Growth, Health status, *Litopenaeus vannamei*, Probiotics

## Abstract

In recent decades, probiotics have become an acceptable aquaculture strategy for shrimp growth promotion and immune modulation. This study aimed to evaluate the effect of *Bacillus velezensis* on *Litopenaeus vannamei* following a 60-day trial. *L. vannamei* (3 ± 0.4 g) were distributed into four groups with three replicates per group and fed an isonitrogenous diet supplemented with *B. velezensis* at 0, 1 × 10^7^, 1 × 10^8^, and 1 × 10^9^ CFU/g, which were defined as the control, G1, G2, and G3 groups, respectively. *B. velezensis* significantly improved the growth, survival rate, and proximate body composition of *L. vannamei* (*P* < 0.05). All groups fed the *B. velezensis* diet showed significant increases in digestive enzymes (lipase, amylase, and protease), superoxide dismutase (SOD; G3), catalase (CAT; G3, G2, and G1), lysozyme activity (G3 and G2), immunoglobulin M (IgM), bactericidal activity BA%, alkaline phosphatase (AKP), and acid phosphatase (ACP) compared with the control group (*P* < 0.05). Malondialdehyde (MDA), triglycerides, cholesterol, high-density lipoprotein (HDL), low-density lipoprotein (LDL), aspartate aminotransferase (AST), and alanine aminotransferase (ALT) levels were significantly decreased in all groups fed *B. velezensis* diet compared with the control group (*P* < 0.05). The expression levels of SOD (G3)*,* LZM*,* and serine proteinase genes were significantly higher in *L. vannamei* fed diets containing *B. velezensis* than in the control group (*P* < 0.05). This is the first study to address the effects of *B. velezensis* on the expression of the LZM and serine proteinase genes in *L. vannamei. L. vannamei* fed diet containing *B. velezensis* had more B and R cells in its hepatopancreas than did the control group. In conclusion, *B. velezensis* is a promising probiotic that can be safely added to the diet of *L. vannamei* with 1 × 10^9^ CFU/g. Its application had a positive influence on the health status, survival rate, nutritional value, and immunity of *L. vannamei*.

## Introduction

Shrimp farming, particularly of *Litopenaeus vannamei*, is increasing significantly in many countries throughout the world [1–3], including Egypt, which has recently taken on the challenge of achieving self-sufficiency in fish and fish products through megaprojects like Ghalioun and Sharq Al-Tafria.

*Litopenaeus vannamei*, also known as Vannamei shrimp, *Penaeus vannamei*, Pacific white shrimp, and whiteleg shrimp, is a shrimp species that efficiently utilises the natural productivity of its ponds, even under intensive culture conditions. Furthermore, it has lower feed costs due to its lower protein requirement (18–35%) compared to more carnivorous shrimp species (36–42%) [4] and is considered a major commercial food commodity due to its high price, high resistance to disease, excellent flavour, great nutritional value, well-understood aquaculture technologies, and vast consumption [5–7].

Farming intensification practices and climatic changes during *L. vannamei* aquaculture increase its susceptibility to different types of diseases, leading to great economic losses, and the demand for environmentally friendly alternatives to antibiotics is critical. Probiotics (non-pathogenic bacteria) are a strategy used to improve shrimp aquaculture by promoting digestion and disease resistance. They can also be employed as antibiotic alternatives, thereby preventing drug resistance to routinely used antimicrobials and antibiotic residues [5, 8].

Probiotics play a major role in fish and shellfish aquaculture productivity. They positively impact survival and growth rates, preserve water quality in ponds, and aid in the digestion and absorption of nutrients. The above reasons led to higher growth performance and a higher feed conversion ratio [9–11]. They improve the innate and adaptive immune responses of shrimp, resulting in increased resistance to infectious diseases through increased hemocyte counts, phenoloxidase activity, respiratory bursts, and the upregulation of antimicrobial gene expression. They also improve antioxidant enzymes, such as SOD&CAT, and decrease oxidative stress marker e.g. MDA which in turn makes the incorporation of probiotics a key benefit in shrimp aquaculture [12–14].

*Bacillus* spp., *Lactobacillus* spp., *Pediococcus* spp., and *Enterococcus* spp. are among the bacterial species employed as probiotics in aquaculture. *Bacillus* spp. are excellent examples of probiotics with superior properties, such as the production of non-pathogenic and non-toxic antipathogenic materials and the ability to produce spores, which extend the shelf life and make them more resistant to adverse environmental conditions [15–21].

Despite the use of *Bacillus* spp. in aquaculture of different aquatic species e.g. *B. subtilis* in *L.* *vannamei*, *B. velezensis* in grass carp, and *B. amyloliquefaciens* in *Oreochromis niloticus* [22–24], studies on *B. velezensis* in shrimp diets are scarce [14, 25]. Therefore, this study aimed to assess the impact of this probiotic on *L.* *vannamei* growth performance, survival rate, feed utilisation, body composition, biochemical parameters, antioxidants, and immune-related gene expression, in addition to histopathological alterations.

## Material and methods

The current study followed a standard working methodology approved by the Animal Use and Care Committee of the Faculty of Fish and Fisheries Technology at Aswan University, Egypt (Protocol No. 5/2022).

### The probiotic used and its safety

*B. velezensis* used in this study was a locally isolated Egyptian strain obtained from a healthy fish pond (National Research Center Project No. 12050419–2019, Egypt). It was genetically identified as *B. velezensis* (unpublished data).

In fibreglass tanks, 120 white shrimp with an average initial body weight of 3 ± 0.4 g/shrimp were randomly divided into four groups in triplicate (10 shrimp per tank) to test the safety of used *B. velezensis* for this shrimp species. Diets supplemented with four different levels of *B. velezensis* (CG: 0 CFU/g as a control group; G1: 1 × 10^7^ CFU/g; G2: 1 × 10^8^ CFU/g; and G3: 1 × 10^9^ CFU/g) were fed to *L.* *vannamei* ad libitum according to García-Bernal et al. [26]. Activity, behavior, and survival rates of *L. vannamei* were checked daily for 3 days.

### Diets preparation

A commercial isonitrogenous basal diet specifically formulated for shrimp from a factory in Kafer Elsheikh, Egypt was used in this study. The ingredients and chemical analysis of the basal diet is shown in Table 1. According to Cunniff and AOAC [27], moisture (7.45%), crude protein (40.28%), crude fat (6.85%), and crude ash (9.84%) were present. Moisture content was measured by drying the diet sample in a hot-air oven at 135 °C then gets the difference between the sample weight before and after drying. Protein was measured using the Kjeldahl method, in which sulfuric acid was used for digestion, copper sulfate was used as a catalyst, and ammonia was finally titrated. The Soxhlet apparatus was used to determine the crude lipid content with chloroform–methanol extraction. Gross energy was measured using a bomb calorimeter. *B. velezensis* was added to the basic diet according to Toften and Jobling [28] and stored at 4°C until use. In summary, *B. velezensis* was mixed with sterile saltwater to form suspensions. Diets were prepared by sprinkling the feed with water suspensions containing *B. velezensis* at different concentrations according to Chen et al. [25] (1 × 107 CFU/g, 1 × 108 CFU/g, and G3: 1 × 109 CFU/g), mixing well, and allowing the feed to dry in a cool dry place. An equivalent quantity of sterile saltwater was added to the diet of the control group. The plate count method using tryptic soyagar plates was used to verify the bacterial load in the diet. To ensure high probiotic levels in the supplemental diet, it was made on a weekly basis.

**Table 1:** Ingredients and chemical analysis of the experimental diet on a dry matter basis

Components	Per 100 g diet
Wheat bran	26.4
Soybean meal- CP 48%	22.4
Poultry meat meal	16.6
Fish meal	16.3
Rice polishing (fine bran)	6.64
Gluten	6
Distillers grains	3.2
Phospholipids	1.2
Monocalcium phosphate	0.5
Shrimp vitamin and Mineral premix^a^	0.3
Vit. C	0.26
Pellet binder	0.1
Anti-fungal (Silicate)	0.1
Proximate chemical analysis g/kg diet	
Moisture	7.45
Crude fiber	4.93
Crude protein	40.28
Crude lipid	6.85
NFE^b^	38.1
Ash	9.84
GE (MJ/kg diet)^c^	11.5

^a^Vitamin and mineral mixture each 1 kg of mixture contains 4800 IU vitamin A, 2400 IU cholecalciferol (vitamin D), 40 g vitamin E, 8 g vitamin K, 4.0 g vitamin B12, 4.0 g vitamin B2, 6 g vitamin B6, 4.0 g pantothenic acid, 8.0 g nicotinic acid, 400 mg folic acid, 20 mg biotin, 200 gm choline, 4 g copper, 0.4 g iodine, 12 g iron, 22 g manganese, 22 g zinc, 0.04 g selenium, 1.2 mg folic acid, 12 mg niacin, 26 mg D-calcium pantothenate, 6 mg pyridoxine HCl, 7.2 mg ribo ￡ avin, 1.2 mg thiamine HCl, 3077 mg sodium chloride (NaCl, 39% Na, 61% Cl), 65 mg ferrous sulfate (FeSO4 ・ 7H2O, 20% Fe), 89 mg manganese sulfate(MnSO4, 36% Mn), 150 mg zinc sulfate (ZnSO4. 7H2O, 40% Zn), 28 mg copper sulfate (CuSO4 5H2O, 25% Cu), 11 mg potassium iodide (KI, 24% K, 76% I), 1000 mg Celite AW521 (acid-washed diatomaceous earth silica). W% on a dry matter (DM) basis

^b^Nitrogen-free extract (calculated by difference) = 100 − (protein + lipid + ash + fiber)

^c^Gross energy was measured using Bomb Calorimeter, Parr 1356 Bomb Calorie

### Experimental design

Obviously healthy 500 *L. vannamei,* nearly of the same size, were obtained from a private farm in Port Said Governorate, Egypt, (Elshamy farm), where the research was conducted. Informed consent was obtained from the farm’s owner before conducting the trial on the shrimp. Shrimp were acclimatized for 2 weeks in outdoor 1000-L fibreglass tanks and fed a basal diet without probiotics. After adaptation, 300 shrimp with an average initial body weight of 3 ± 0.4 g/shrimp were randomly allocated into four groups in triplicate in 1 m^3^ fibreglass tanks (25 shrimp per tank). *L.* *vannamei* fed diets supplemented with four different levels of *B. velezensis* (CG: 0 CFU/g as a control group; G1: 1 × 10^7^ CFU/g; G2: 1 × 10^8^ CFU/g; and G3: 1 × 10^9^ CFU/g) for 60 successive days as a feeding trial. Shrimp were fed a diet at 6% of its weight their weight within the first 30 days and 4.5% within the next 30 days. Feeding rates were altered every two weeks as shrimp body weights changed. The provided feed was divided into four equal portions and given to the shrimp four times per day (06:00, 12:00, 17:00, and 22:00). Every other day, over 20% of the tank water was exchanged. The cumulative mortality rate of shrimp was recorded daily. Water quality metrics were measured using HANNA instruments (model HI 9829 – Multiparameter, USA) and maintained throughout the study period at: temperature, 27.5–31.1 °C; pH, 8–8.4; dissolved oxygen, 5.1–6.2 ppm; and salinity, 39.9–41.7 ppt. HANNA instrument 733 Ammonia High Range was used to measure ammonia to be 0–0.01 ppm during the study period. Four times a day, the tanks were cleared of unfed diets and shrimp poop.

### Extraction of hemolymph, serum and plasma from *L. vannamei*

Hemolymphs were collected from 5 *L. Vannamei* in each group. Individual shrimp hemolymph (100 μL) was extracted from the pleopod base of the first abdominal segment using a sterile 1-mL syringe (25 G × 13 mm needle) without anticoagulant. Next, the hemolymph was refrigerated at 4°C for 2 h before centrifugation at 10,000 rpm for 15 min to obtain serum. The serum was kept at -20 °C for further examination [29].

Another sample of hemolymph from 5 *L. vannamei* / group was withdrawn in a sterile 1-mL syringe loaded with a precooled (4 °C) solution (SIC-EDTA, Na2) (450 mM NaCl, 10 mM KCl, 10 mM hepes, and 10 mM EDTA, Na2 at pH 7.3) as an anticoagulant [30]. Individual eppendorf tubes were used to retain the hemolymph, which was kept on ice to separate the plasma. Samples of hemolymph were immediately centrifuged at 800 rpm for 10 min at 4 °C, and the plasma was frozen at − 80 °C.

### Growth performance and survival rate

After the feeding trial, the remaining *L. vannamei* were counted, and all growth parameters, such as initial body weight (g), final body weight (FBW), weight gain rate (WG%), specific growth rate (SGR), feed conversion rate (FCR), and survival rate (SR), were calculated as follows, according to Tekinay and Davies [31]:

$$\text{Weight gain rate }(\%) = (\text{FBW} - \text{IBW}) \times 100/\text{IBW}$$

$$\text{Specific growth rate}\ (\text{SGR}) (\%\text{day} ^{-1}) = \text{ln}\ (\text{final mean weight}) - \text{ln}\ (\text{initial mean weight}) / \text{experimental days x}\ 100$$

$$\text{Feed conversion ratio }(\text{FCR}) =\text{ Feed intake }(\text{g}) / \text{ Weight gain }(\text{g}).$$

$$\text{Survival rate}\ (\text{SR}) = (\text{Survival number of shrimp} / \text{ Initial number of shrimp}) \times 100.$$

### Determination of proximate body composition of *L. vannamei*

Following a 60-day feeding study, five randomly selected *L. vannamei*, virtually identical in size, were held at − 20℃ to assess their contents of moisture, dry matter DM, protein, lipid, growth energy GE (Kcal/g), and ash, as per Cunniff and AOAC [27].

### Digestive enzyme activity

The Gastrointestinal tissues were homogenized in cold PBS. GIT homogenate of *L. vannamei* was centrifuged at 18,894 rpm at 4 °C for 5 min, and the supernatant was carefully separated to analyze different digestive enzymes. Lipase was determined using kits from Spectrum Company for Biotechnology, Egypt, slightly modifying the procedure outlined by Moss and Henderson [32]. To put it briefly, lipase splits a synthetic substrate (DGMRE) to produce the colorful end product, methylresorufin. The increasing absorbance of red methylresorufin was measured colorimetrically at a wavelength of 580 nm [32]. Amylase was determined using starch as substrate; in which 2 flasks (test and control) were used. In each flask, 5 ml of starch was added and placed in a water bath at 37 °C for 5 min. In the test flask, 0.1 ml of enzyme extract was added and mixed well, whereas no addition was made in the control flask; the mixture was mixed well and left for 7.5 min. The 2 flasks removed from water bath and added 5 ml of working iodine solution to each flask, diluted to 50 ml with water, and mixed well. At a wavelength of 660 nm, the result was measured colorimetrically [33]. Proteases were determined using a protease activity assay kit (Fluorometric—Green) (ab112152) (Abcam Co, UK), in which casein conjugate served as a generic substrate. A green fluorescent dye was used to label the casein, which significantly quenched the fluorescence. Fluorescence intensity increased in a direct proportion with protease activity. With the FITC filter set, the signal was easily observed at Ex/Em = 490/525 nm using a fluorescent microplate reader [34].

### Antioxidant, immune response and biochemical parameters

A bead homogenizer was used to homogenize several samples, each comprising one part of the hepatopancreas and nine parts of 0.9% saline, in an ice-filled container for 10 min. After centrifugation for 10 min at 4 °C and 3,500 rpm, the supernatant was collected and stored at -80 °C to be used for the detection of superoxide dismutase (SOD), catalase CAT, and lipid peroxide [11]. According to Nishikimi et al. [35], Satoh [36], and Aebi [37], superoxide dismutase (SOD), lipid peroxide (Malondialdehyde) (MDA), and catalase were assessed colorimetrically using kits from Biodiagnostic Co., Egypt at wave lengths of 560, 510, and 534 nm, respectively.

Following the manufacturer’s instructions, a fish lysozyme ELISA kit (Sunlong Biotech Co., China) was used to measure serum lysozyme activity using the ELISA micro-well technique at a wavelength of 450 nm using a microplate ELISA reader. Using a commercial kit (Sunlong Biotech Co., China) and the manufacturer’s instructions, immunoglobulin M (IgM) was quantified by ELISA.

Iida et al. [38] provided a methodology for determining the bactericidal activity BA%, which involved diluting serum samples three, four, and five times in Tris buffer (pH 7.5). The diluted solutions were treated with a bacterial solution (0.001 g/mL, *Aeromonas hydrophila*) for 24 h at 25 °C. Reaction solutions in 50 µL were incubated on TSA for a whole day at 25 °C. The colony-forming unit (CFU) was computed using the plate counting method. A survival index (SI) was created using the data [39] according to the following formula:

$$\text{SI}=\frac{\text{Final CFU}}{\text{Initial CFU}}\times 100$$

Department of Fish Health and Management, Central Laboratory for Aquaculture Research, Egypt kindly provided the *Aeromonas hydrophila* strain (1.7 × 10^6^ CFU) for the serum bactericidal activity test.

From the collected *L. vannamei* serum, aspartate aminotransferase (AST) and alanine aminotransferase (ALT) levels were measured using kits (Biodiagnostic Co., Egypt) according to Reitman and Frankel [40]. Triglycerides, cholesterol, HDL-cholesterol, and LDL-cholesterol were also tested using kits from Biodiagnostic Co., Egypt, according to Fossati and Prencipe [41], Richmond [42], Burstein et al. [43], and Wieland and Seidel [44], respectively.

Using kits from Biodiagnostic Co., Egypt, alkaline phosphatase (AKP) and acid phosphatase (ACP) (Biodiagnostic Co., Egypt) were quantified colorimetrically at a wavelength of 510 nm in accordance with Belfield and Goldberg [45] and Kind and King [46], respectively.

### Total RNA extraction, cDNA synthesis, and real-time quantitative PCR analysis of antioxidant and immune-related genes

Total RNA was extracted from 50 mg of *L. vannamei* hepatopancreatic tissues using trizol (iNtRON Biotechnology) according to the manufacturer’s instructions. The integrity of RNA was confirmed using 2% agarose gel electrophoresis with ethidium bromide. The concentration and purity of RNA were determined using a Nanodrop BioDrop spectrophotometer (Biochrom Ltd, Cambridge CB23 6DW, UK) based on the A260/A280 nm ratio. Two μg of RNA sample were reverse transcribed using the ABT 2X RT Mix cDNA synthesis kit according to manufacturer’s Protocol. Gene expression profiling was performed in Rotor Gene-Q (Qiagen-Germany) using gene-specific primer sequences for the amplification of the antioxidation-related gene (SOD) and immune genes serine proteinase and lysozyme (LZM) genes (Table 2). The amplification reaction was performed using an ABT 2X qPCR Mix (SYBR) kit. The reaction volumes was 20 μl consisting of 10 µL SYBR Green, 0.6 µL of forward and reverse specific primers, 1 µL of cDNA template, and nuclease-free water, to make a final volume 20 µL. The PCR program was performed with the following conditions: activation at 95 °C for 15 min, followed by 40 cycles of denaturation at 95 °C for 10 s, annealing at the primer-specific temperature for 15 s, and extension at 72 °C for 25 s. This was followed by a melt curve analysis to assess the specificity of amplification at 72 °C to 95 °C. All genes were tested in triplicate. CT values for each sample were determined and incorporated into “fold change” (2 − ΔΔCT), calculation based on Livak and Schmittgen [47], and mRNA expressions for each sample were normalized against beta actin as a housekeeping gene.

**Table 2:** Primer sequences of antioxidant and immune-related genes in *Litopenaeus vannamei* fed *Bacillus velezensis*- enriched diets for 60 days

Gene	Primer sequence (5′ -3′)	Accession No.	Slope	Efficiency %	Amplified product size (bp)	Reference
Internal reference gene (*β-actin*)	F: CCA CGA GAC CAC CTA CAA C R: AGC GAG GGC AGT GAT TTC	AF300705	- 3.31	100.50	142	Flores-Miranda et al. [48]; Fierro Coronado et al. [49]; Wang et al. [50]
Superoxide dismutase (*SOD*)	F: ATC CAC CAC ACA AAG CAT CA R: AGC TCT CGT CAA TGG CTT GT	DQ005531	- 3.401	96.80	229	Han-Ching et al. [51]
Serine proteinase	F: CGT CGT TAG GTT AAG TGC GTT CT R: TTT CAG CGC ATT AAG ACG TGT T	AY368151.1	- 3.33	99.66	61	Jiménez-Vega et al. [52]; Zokaeifar et al. [53]
Lysozyme (*LZM*)	F: GAA GCG ACT ACG GCA AGA AC R:AAC CGT GAG ACC AGC ACT CT	AF425673	- 3.39	97.24	216	Han-Ching et al. [51]

### Histological examination

*L. vannamei* hepatopancreas (3 samples) were collected from each group, fixed for 24 h in Davidson’s fixative, and then transferred to 70% ethanol. Subsequently, they were passed through ascending grades of ethyl alcohol for dehydration and then cleared in xylene. Cleared tissues were embedded in paraffin wax. The paraffin blocks were sectioned into 5µm-thick sections and stained with H&E according to Bell and Lightner [54]. The numbers of E, B, and R cells were counted using ImageJ software with a cell counter plugin in 20 randomly chosen tubules from each treatment according to Abd El-Naby et al. [34].

### Statistical analysis

The data were subjected to one-way ANOVA using IBM SPSS Statistics (Version 22), and the results were displayed as mean ± standard error (SE). Significant differences between the examined groups were determined using a multiple-range test [55]. Differences between groups were considered statistically significant at *P* < 0.05. For each parameter, values in the same row with distinct superscript letters (a, b, c, and d) indicate significantly different mean values.

## Results

### *Bacillus velezensis* safety

The application of different concentrations of *B. velezensis* in *L. vannamei* diets did not cause any abnormal activity, behavior, or mortality in this shrimp species.

### Growth performance, proximate body composition and digestive enzymes

The growth performance and survival rate of test diets fed to *L. vannamei* and supplemented with varying amounts of *B. velezensis* for 60 days are presented in Table 3. Although only the G3 and G2 groups showed a significant increase in the specific growth rate compared with the control, all *B. velezensis*-supplemented groups had significantly higher final body weight, weight gain rate, and survival rate. Compared with the control group fed a diet free of *B. velezensis*, the *L. vannamei* feed conversion ratio was significantly improved after *B. velezensis* supplementation. Among the supplemented groups, *L. vannamei* fed a diet containing 1 × 10^9^ CFU/g of *B. velezensis* exhibited the best overall growth performance (final body weight, weight gain rate, specific growth rate, and feed conversion ratio).

**Table 3:** Growth performance and survival rate of *Litopenaeus vannamei* fed test diets supplemented with different levels of *Bacillus velezensis* for 60 days

Item	*Bacillus velezensis* level (CFU / g diet)			
	0 (CG)	1 × 10^7^ (G1)	1 × 10^8^ (G2)	1 × 10^9^ (G3)
Initial body weight (g)	3.14 ± 0.07	3.14 ± 0.13	3.2 ± 0.06	3.27 ± 0.08
Final body weight (g)	13.93 ± 0.46^c^	15.83 ± 0.26^b^	16.47 ± 0.12^b^	17.37 ± 0.09^a^
Weight gain Rate (%)	346.22 ± 1.63^d^	403.41 ± 1.36^c^	418.25 ± 2.0^b^	437.79 ± 3.33^a^
Specific growth rate (SGR) %/day	2.48 ± 0.07^b^	2.65 ± 0.06^ab^	2.73 ± 0.04^a^	2.79 ± 0.03^a^
Feed conversion ratio (FCR) (g FI/g WG)	2.12 ± 0.06^a^	1.96 ± 0.35^b^	1.78 ± 0.06^c^	1.62 ± 0.04^c^
Survival rate (SR) (%)	84 ± 2.3^b^	90.67 ± 1.33^a^	92 ± 2.31^a^	93.33 ± 1.33^a^

Values expressed as means ± SE (*n* = 3). Different superscript letters indicate significant differences for each pairwise comparison between treatments (*P* < 0.05)

CG 0 CFU/g (control group), G1 1 × 10^7^ CFU/g (group 1), G2 1 × 10^8^ CFU/g (group 2), G3 1 × 10^9^ CFU/g (group 3), *IBW* initial body weight, *FI* feed intake, *WG* weight gain

The proximate body composition of *L. vannamei-*fed test diets supplemented with varying amounts of *B. velezensis* for 60 days was significantly improved compared to the control. All groups fed the studied diets, especially G3, showed significant increases (*P* < 0.05) in DM, protein, and GE compared with the control group. The *B. velezensis*-supplemented diet groups had considerably lower moisture, fat content, and ash content than the control group (*P* < 0.05) (Table 4).

**Table 4:** Whole body composition of *Litopenaeus vannamei* fed test diets supplemented with different levels of *Bacillus velezensis* for 60 days on wet basis

Items	*Bacillus velezensis* level (CFU / g diet)			
	0 (CG)	1 × 10^7^ (G1)	1 × 10^8^ (G2)	1 × 10^9^ (G3)
Moisture %	74.57 ± 0.40^a^	67.93 ± 0.12^c^	69.17 ± 0.44^b^	66.01 ± 0.05^d^
DM %	25.73 ± 0.21^c^	32.74 ± 0.22^b^	32.17 ± 0.44^b^	34.35 ± 0.32^a^
Protein %	16.48 ± 0.45^c^	23.37 ± 0.33^b^	23.25 ± 0.56^b^	26.2 ± 0.39^a^
EE %	3.74 ± 0.07^a^	3.23 ± 0.06^b^	2.93 ± 0.06^c^	2.52 ± 0.05^d^
Ash %	4.83 ± 0.03^a^	4.24 ± 0.04^c^	4.66 ± 0.03^b^	3.84 ± 0.03^d^
GE Kcal/g	1.49 ± 0.05^c^	2.12 ± 0.08^b^	2.26 ± 0.05^b^	2.75 ± 0.04^a^

Values expressed as means ± SE (*n* = 3). Different superscript letters indicate significant differences for each pairwise comparison between treatments (*P* < 0.05)

CG 0 CFU/g (control group), G1 1 × 10^7^ CFU/g (group 1), G2 1 × 10^8^ CFU/g (group 2), G3 1 × 10^9^ CFU/g (group 3)

All groups fed *B. velezensis* diets showed a significant (*P* < 0.05) improvement in the digestive enzymes of *L. vannamei* GIT (lipase, amylase, and protease) compared with the control group; G3 was the highest in all groups (Table 5).

**Table 5:** Digestive enzymes activity of *Litopenaeus vannamei* fed test diets supplemented with different levels of *Bacillus velezensis* for 60 days

Items	*Bacillus velezensis* level (CFU / g diet)			
	0 (CG)	1 × 10^7^ (G1)	1 × 10^8^ (G2)	1 × 10^9^ (G3)
Lipase U/L	30.83 ± 0.38^d^	44.35 ± 0.73^b^	38.97 ± 0.50^c^	46.98 ± 0.63^a^
Amylase U/L	20.49 ± 0.51^d^	23.61 ± 0.35^c^	25.99 ± 0.41^b^	29.97 ± 0.18^a^
Protease (ng/mg)	55.04 ± 2.5^c^	86.26 ± 0.79^b^	113.88 ± 7.98^a^	127.19 ± 1.53^a^

Values expressed as means ± SE (*n* = 3). Different superscript letters indicate significant differences for each pairwise comparison between treatments (*P* < 0.05)

CG 0 CFU/g (control group), G1 1 × 10^7^ CFU/g (group 1), G2 1 × 10^8^ CFU/g (group 2), G3 1 × 10^9^ CFU/g (group 3)

### Antioxidant enzymes and immune variables

The antioxidant enzyme SOD was significantly enhanced (*P* < 0.05) in G3 only compared with the other groups; while CAT showed a significant increase (*P* < 0.05) in all groups fed the BV diet compared with the control group. MDA showed a significant decrease (*P* < 0.05) in all groups fed the *B. velezensis* diet compared with the control group, especially G3, G2, and G1 in succession.

The lysozyme activities of G3 and G2 were significantly higher (*P* < 0.05) than those of G1 and the control group as immune response biomarkers. Compared with the control group, all groups fed *B. velezensis* diets had a substantial increase (*P* < 0.05) in IgM, with G2 exhibiting the highest value. In all groups fed *B. velezensis* diets, BA% increased significantly (*P* < 0.05) compared with the control group (Table 6).

**Table 6:** Antioxidants and immune response parameters of *Litopenaeus vannamei* fed test diets supplemented with different levels of *Bacillus velezensis* for 60 days

Items	*Bacillus velezensis* level (CFU / g diet)			
	0 (CG)	1 × 10^7^ (G1)	1 × 10^8^ (G2)	1 × 10^9^ (G3)
SOD U/g	10.37 ± 0.49^b^	9.96 ± 0.31^b^	10.23 ± 0.23^b^	13.96 ± 0.23^a^
CAT U/g	6.68 ± 0.28^d^	9.33 ± 0.37^c^	15.91 ± 0.56^a^	12.87 ± 0.36^b^
MDA nmol/g	28.97 ± 0.09^a^	22.82 ± 0.22^b^	17.13 ± 0.44^c^	15.22 ± 0.77^d^
Lysozyme activity	4.4 ± 0.26^c^	4.44 ± 0.28^c^	6.03 ± 0.41^b^	7.07 ± 0.03^a^
IgM µg/ml	2.6 ± 0.05^d^	3.14 ± 0.09^c^	4.4 ± 0.3^a^	3.51 ± 0.13^b^
BA %	50.03 ± 0.54^d^	55.5 ± 0.2^a^	52.42 ± 0.56^b^	51.1 ± 0.74^c^

Values expressed as means ± SE (*n* = 3). Different superscript letters indicate significant differences for each pairwise comparison between treatments (*P* < 0.05)

CG 0 CFU/g (control group), G1 1 × 10^7^ CFU/g (group 1), G2 1 × 10^8^ CFU/g (group 2), G3 1 × 10^9^ CFU/g (group 3)

### Biochemical parameters

The addition of dietary *B. velezensis* at varying concentrations to the diet of *L. vannamei* resulted in a significant decrease in the activity of enzymes linked to hepatopancreatic function, including AST and ALT. AKP and ACP levels were significantly increased in all groups fed the test diets compared with the control group (Table 7).

**Table 7:** Biochemical parameters of *Litopenaeus vannamei* fed test diets supplemented with different levels of *Bacillus velezensis* for 60 days

Items	*Bacillus velezensis* level (CFU / g diet)			
	0 (CG)	1 × 10^7^ (G1)	1 × 10^8^ (G2)	1 × 10^9^ (G3)
AST U/L	17.4 ± 0.31^a^	12.4 ± 0.31^c^	15.53 ± 0.23^b^	15.33 ± 0.29^b^
ALT U/L	43.67 ± 0.5^a^	34.3 ± 0.25^b^	20.68 ± 0.68^d^	24.73 ± 0.43^c^
AKP U/L	25.63 ± 0.64^d^	31.3 ± 0.15^c^	49.88 ± 0.75^b^	64.19 ± 0.58^a^
ACP U/L	15.63 ± 0.2^c^	18.77 ± 0.37^b^	19.53 ± 0.94^b^	20.5 ± 0.23^a^

Values expressed as means ± SE (*n* = 3). Different superscript letters indicate significant differences for each pairwise comparison between treatments (*P* < 0.05)

*AKP *Alkaline phosphatase, *ACP* Acid phosphatase, CG 0 CFU/g (control group), G1 1 × 10^7^ CFU/g (group 1), G2 1 × 10^8^ CFU/g (group 2), G3 1 × 10^9^ CFU/g (group 3)

### Lipids profile

Regarding the lipid profile presented in Table 8, all groups of *L. vannamei* fed *B. velezensis* diets had a significant (*P* < 0.05) decreases in triglycerides, cholesterol, high-density lipoprotein, and low-density lipoprotein compared with the control group. A dose-dependent decrease in cholesterol and triglyceride levels was observed.

**Table 8:** Lipid profile of *Litopenaeus vannamei* fed test diets supplemented with different levels of *Bacillus velezensis* for 60 days

Items	*Bacillus velezensis* level (CFU / g diet)			
	0 (CG)	1 × 10^7^ (G1)	1 × 10^8^ (G2)	1 × 10^9^ (G3)
Triglycerides mg/dL	73 ± 0.58^a^	71.67 ± 0.33^b^	56 ± 0.57^c^	43 ± 0.58^d^
Cholesterol mg/dL	45.67 ± 0.33^a^	43.33 ± 0.33^b^	42 ± 0.58^c^	38.33 ± 0.88^d^
HDL^a^-c mg/dL	20.58 ± 0.68^a^	19.67 ± 0.33^b^	19.33 ± 0.67^b^	17.33 ± 0.33^c^
LDL^b^-c mg/dL	12.4 ± 0.72^a^	10.8 ± 0.4^c^	11.73 ± 0.75^b^	9 ± 0.46^d^

Values expressed as means ± SE (*n* = 3). Different superscript letters indicate significant differences for each pairwise comparison between treatments (*P* < 0.05)

CG 0 CFU/g (control group), G1 1 × 10^7^ CFU/g (group 1), G2 1 × 10^8^ CFU/g (group 2), G3 1 × 10^9^ CFU/g (group 3)

^a^HDL-c: High density lipoprotein

^b^LDL-c: Low density lipoprotein

### Hepatopancreatic antioxidant

**Fig. 1:**
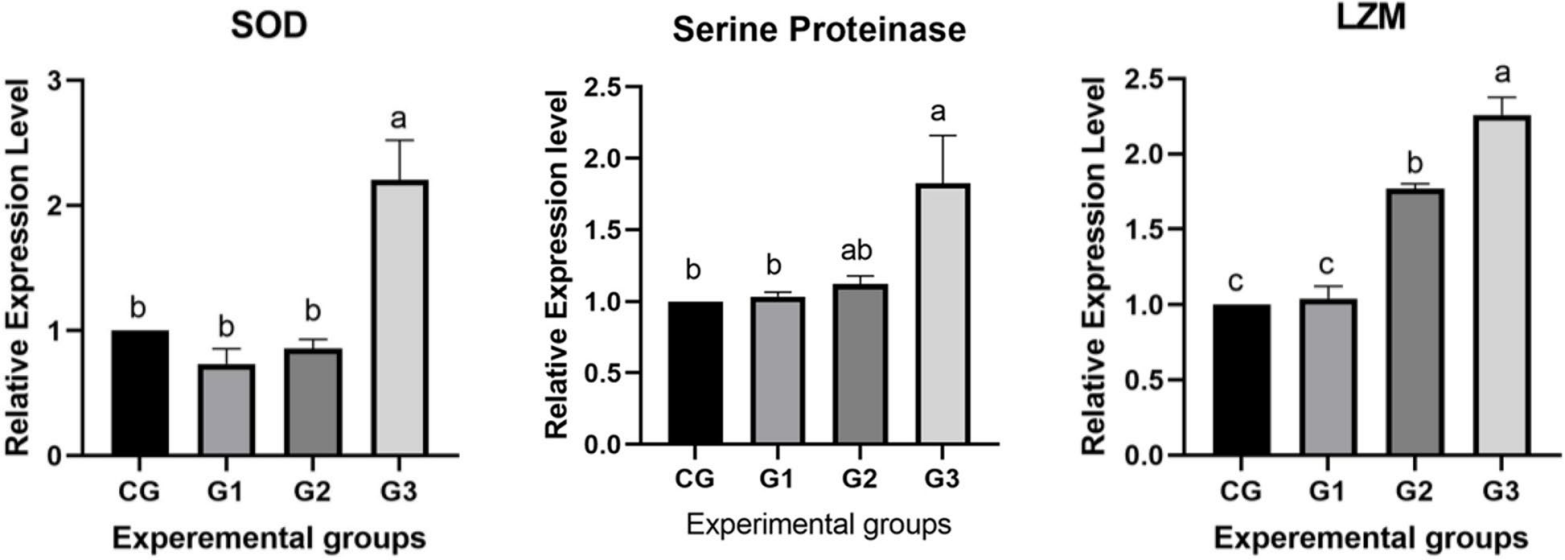
Relative expression of antioxidative gene (*SOD*) and immune related gene expression (*LZM* and serine proteinase) of *Litopenaeus vannamei* fed test diets supplemented with *Bacillus velezensis* of 0 CFU/g as (CG; a control group); 1 × 10^7^ CFU/g (G1); 1 × 10^8^ CFU/g (G2); and 1 × 10^9^ CFU/g (G3) for 60 days. Values are expressed as mean ± SE from triplicate groups. Bars with different letters are significantly different from those of control group (*P* < 0.05). CG: 0 CFU/g (control group); G1: 1 × 10^7^ CFU/g (group 1); G2: 1 × 10^8^ CFU/g (group 2); G3: 1 × 10.^9^ CFU/g (group 3)

Hepatopancreatic antioxidant gene expression (Fig. 1) showed no significant difference in SOD mRNA levels between the control, G1, and G2 groups. However, G3 cells exhibited significantly higher (*P* < 0.05) SOD expression compared to these groups. On the other hand, in the hepatopancreatic immune response for *LZM* and serine proteinase genes, G3 had the highest expression level, followed by G2, whereas G1 lacked a statistically significant difference in gene expression compared with the control group. Interestingly, in this study, the hepatopancreatic SOD and serum lysozyme activities using the diagnostic kits, as well as the transcript levels of these genes in the hepatopancreas, were identical.

### Histological examination

**Fig.2:**
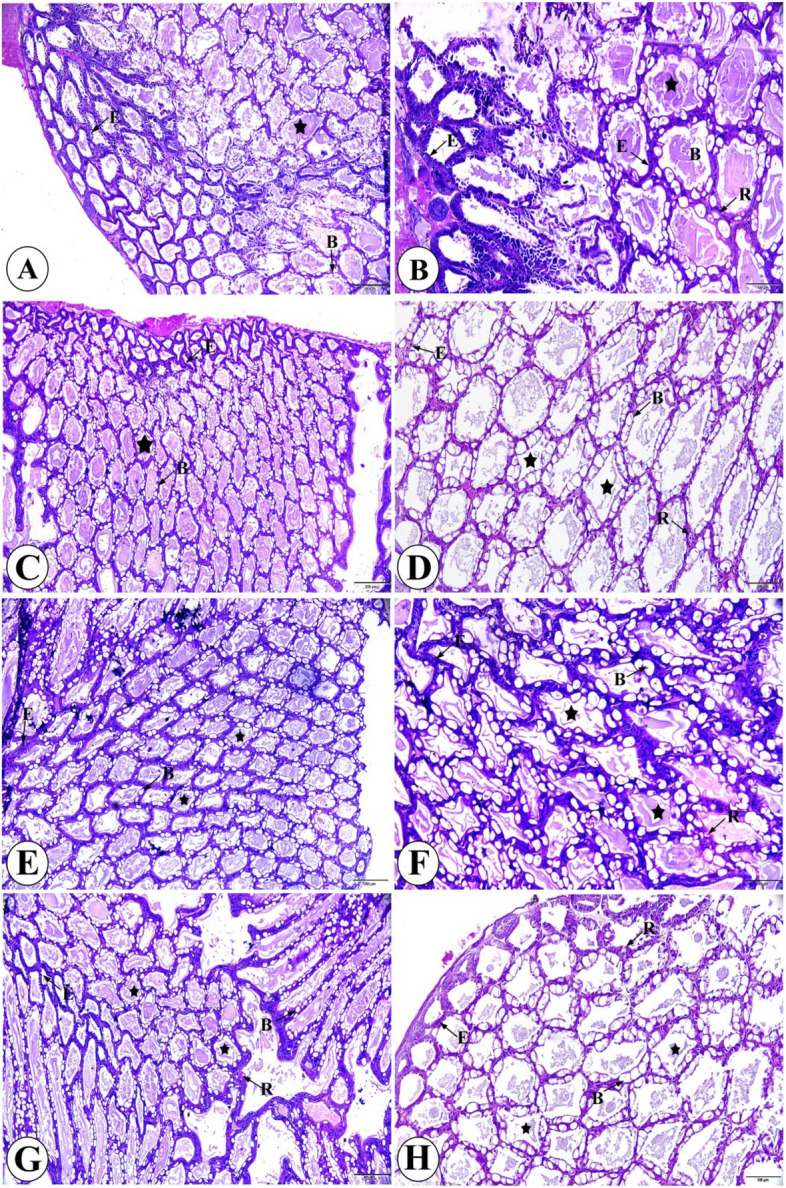
Histomicrograph of hepatopancreas of *Litopenaeus vannamei*: **A** & **B** Control group (no *Bacillus velezensis*) showing many strongly basophilic cuboidal embryonic cells (E) with large centrally located nuclei. **C** & **D** G1, 1 × 10^7^ CFU/g *Bacillus velezensis*; **E** & **F** G2, 1 × 10^8^ CFU/g *Bacillus velezensis*; **G** & **H** G3, 1 × 10^9^ CFU/g *Bacillus velezensis* for 60 days showing larger globular-shaped vesicular secreting cells (B) with basal nuclei and large apical vacuoles, large reabsorption cells (R) with round basal nuclei, and multivacuolar granular cytoplasm, as well as star-shaped and polygonal liver tubules. **H **& **E** stain, bar of A, C, E, G = 200 µm; bar of B, D, F, H = 100 µm. CG: 0 CFU/g (control group); G1: 1 × 10^7^ CFU/g (group 1); G2: 1 × 10^8^ CFU/g (group 2); G3: 1 × 10.^9^ CFU/g (group 3)

The hepatopancreas of *L. vannamei* fed *B. velezensis*-supplemented diets did not exhibit any histopathologic changes. However, *L. vannamei* fed the control diet showed a significant increase in the number of strongly basophilic cuboidal embryonic cells (E) with large centrally located nuclei compared with the *B. velezensis*-supplemented groups (*P* < 0.05). In contrast, the *B. velezensis*-supplemented groups displayed a significant increase (*P* < 0.05) in large globular-shaped vesicular secreting cells (B) with basal nuclei and large apical vacuoles, large reabsorption cells (R) with round basal nuclei and multivacuolar granular cytoplasm, as well as star-shaped and polygonal liver tubules (Fig. 2 and Table 9).

**Table 9:** Prevalence of E cell, B cell, and R cell (number/tubule) in the hepatopancreas of *Litopenaeus vannamei* fed test diets supplemented with different levels of *Bacillus velezensis* for 60 days

Item	*Bacillus velezensis* level (CFU / g diet)			
	0 (CG)	1 × 10^7^ (G1)	1 × 10^8^ (G2)	1 × 10^9^ (G3)
E cell	20.6 ± 1.09^a^	12.61 ± 0.5^b^	9.1 ± 0.45^c^	8.47 ± 0.56^c^
B cell	3.89 ± 0.62^c^	5.5 ± 0.45^b^	8.34 ± 0.96^a^	8.79 ± 0.5^a^
R cell	47.76 ± 1.78^c^	54.9 ± 2.74^b^	57.77 ± 2.72^b^	66.03 ± 2.26^a^

Values expressed as means ± SE. Different superscript letters indicate significant differences for each pairwise comparison between treatments (*P* < 0.05)

CG 0 CFU/g (control group), G1 1 × 10^7^ CFU/g (group 1), G2 1 × 10^8^ CFU/g (group 2), G3 1 × 10^9^ CFU/g (group 3)

## Discussion

Nowadays, natural compounds like probiotics that boost growth, antioxidant capacity, and immune responses have received much attention in *L. vannamei* aquaculture, particularly in Egypt. Moreover, *B. velezensis* has not received as much attention as other species in its family [21, 23, 53, 56]. Accordingly, this study was interested in the locally isolated probiotic *B. velezensis*, which has been studied as a natural feed additive for *L. vannamei*.

Feeding different doses of *B. velezensis* did not cause mortality in *L. vannamei*, indicating that this strain is safe for the species. Chen et al. [25] and Yang et al. [14] also showed that *B. velezensis* was not toxic to *L. vannamei*.

According to the current study, *L. vannamei* improved feed utilization, survival rate, proximate body composition, digestive enzyme activities, antioxidants, and immune response when fed *B. velezensis*-enriched diets containing 1 × 10^9^ CFU/g for eight weeks which reflected in increased growth performance, WG rate and SGR, as well as decreased FCR compared with the control group. The improved growth performance observed in this study may be a result of *Bacillus* species having been shown to improve the secretion of digestive enzymes, increase the digestibility and absorption of nutrients, increase fish appetite by producing vitamins and organic acids, produce certain essential micronutrients, detoxify harmful compounds in the diet, secrete several metabolites with antimicrobial properties, and suppress pathogenic microorganisms that reduce growth [22, 57–63]. Chen et al. [25] and Zhang et al. [64] reported similar findings in *L. vannamei* and Amur minnow *Rhynchocypris lagowskii*, respectively. Furthermore, Ji et al. [65] reported that *B. velezensis* YFI-E109 contained genes involved in metabolic regulation that facilitate amino acid and carbohydrate transport and metabolism, thereby enhancing hybrid yellow catfish growth.

Several factors influence the proximate body composition of aquatic animals, including age, sex, season, shrimp feed, density, and water quality [66]. All groups fed *B. velezensis* diets demonstrated a significant rise in DM, protein, and GE but lower moisture, fat content, and ash content than the control group. Protein is the most essential biochemical component of shrimp bodies, and its content is influenced by diet digestibility [66]. The differences in body composition between *L. vannamei*-fed *B. velezensis* and the control group indicate improvements in the nutritional value of *L. vannamei*-fed *B. velezensis*.

Digestive enzyme activity can help understand the digestive physiology and nutritional requirements of white shrimp [67]. This study showed that digestive enzymes, including amylase, lipase, and protease, were significantly increased in *L. vannamei* fed *B. velezensis* diets compared with the control group. Increased digestive enzyme levels reflect improved growth performance of *L. vannamei*. Numerous studies have also demonstrated the same result; Yang et al. [14] reported enhancements in lipase and α-amylase activities in *L. vannamei* fed with *B. velezensis* at feed concentrations of 0.3 and 0.4 g/kg. Wang [68] observed similar outcomes in *L. vannamei*-fed probiotics, including *Bacillus* sp. and photosynthetic bacteria. Amoah et al. [17] reported the same results in *L. vannamei*-fed diets supplemented with *B. coagulans*. Probiotic strains, particularly *Bacillus* sp., are renowned for their ability to synthesize a wide array of exoenzymes that improve diet digestibility and increase the burden of beneficial bacteria linked with probiotics used in *L. vannamei* [21, 25]. Therefore, higher enzymatic activity improves digestibility and maximizes feed use.

Reactive oxygen species (ROS) are natural metabolites of ordinary cellular metabolism [69]. They are created and negated at equilibrium; however, any stressor can upset this equilibrium, leading to toxic and harmful effects in live cells [70]. Living cells counteract these effects and preserve their equilibrium via a variety of defensive systems, including antioxidant defense mechanisms [71, 72]. On the other hand, measuring the antioxidant enzymes SOD and CAT can indicate the oxidative stress status and antioxidant capacity of aquatic organisms [18, 73–75]. According to our findings, the G3 group and all other groups fed the *B. velezensis* diet had higher SOD and CAT activities than the control group. This study revealed that the *B. velezensis* diet had stronger hepatopancreatic anti-oxidative effects on *L. vannamei* as those reported by Yang et al. [14] and Chen et al. [25], who observed elevated SOD and CAT levels in *L. vannamei* fed the *B. velezensis* diet. SODs are very effective at reducing oxidative stress because they eliminate excess reactive oxygen species (ROS) by degrading excess superoxide radicals, producing oxygen and hydrogen peroxide, and thus halting free radical damage to cells [76–78]. CAT can eliminate hydrogen peroxide, which is produced by SODs, from cells producing molecular oxygen and water [79]. In the current study, *B. velezensis* exhibited antioxidative activity by upregulating antioxidative gene expression; *SOD* (in G3) in addition to two immune-related genes; Serine proteinase and *LZM* (in G3 and G2) in *L. vannamei*. Similar results were reported by Chen et al. [25], who reported a significant increase in *SOD* expression in *L. vannamei* fed *B. velezensis*- enriched diets at 10^7^ CFU/g (BV2) and 10^9^ CFU/g concentrations. Shrimp depend entirely on innate immune responses against pathogenic infections through cellular and humoral responses, such as prophenoloxidase (proPO) activation, toll pathway initiation, hemolymph coagulation, complement activation, melanization, phagocytosis, encapsulation, and antimicrobial peptide synthesis interceded by serine proteinase cascades [80–82]. Serine proteinase (*SP*) is an essential proteolytic enzyme involved in various physiological processes, including digestion, blood coagulation, embryonic development, and immune response [83]. Thus*, B. velezensis* has a positive impact on the expression of serine proteinase in *L. vannamei,* which improves digestive and immune responses. *LZM* is an antimicrobial peptide that is produced primarily by shrimp hemocytes and plays an important role in the innate immune response of shrimp to various microbial infections [84–86]. *LZM* is mainly expressed in the hepatopancreas [87]. To the best of our knowledge; this is the first study to address the effects of *B. velezensis* on the expression of the serine proteinase and *LZM* genes in *L. vannamei.*

The non-enzymatic antioxidant MDA is a lipid peroxidation product that can detect the body's lipid oxidative stress [87, 88]. The hepatopancreatic MDA level of *L. vannamei*-fed *B. velezensis* diets was much lower than that of shrimp fed the control diet, indicating that *B. velezensis* did not cause oxidative stress and may even reduce it. In accordance, Amoah et al. [17] found decreased levels of MDA in *L. vannamei*-fed *B. coagulans*-enriched diets.

Lysozyme is a functional antimicrobial protein and key component of shrimp innate immunity that contributes significantly to host responses to infectious agents [7, 89–92]. Lysozyme activity in *L. vannamei*-fed *B. velezensis* diets was significantly higher in G3 and G2 compared to G1 and the control group. Similarly, prior studies found that *L. vannamei* fed *B. coagulans*-enriched diets exhibited increased lysozyme activity compared with the control diet [17].

Shrimp lacks an acquired immune system and relies mostly on innate immunological responses via cellular and humoral mechanisms [93–95]. The primary humoral reaction involves IgM release to destroy invading microorganisms. Our study found a rise in IgM levels in *L. vannamei* fed *B. velezensis* diets, indicating an enhanced immunological state. In fish, IgM is the first antibody generated during infection and plays a crucial role in systemic and mucosal immune tissues [96]. *B. velezensis* enhances IgM gene expression in grass carp [28]. Similarly, *B. velezensis* was among the three Bacillus species that increased IgM levels in *Oreochromis niloticus* (Kuebutornye et al. 2020). *Rhynchocypris lagowskii* fed on *B. velezensis* had elevated IgM levels [64].

In this study, *B. velezensis* exhibited potent bactericidal activities compared with the control group, which modulated the immune response of *L. vannamei*, thereby increasing disease resistance. This could be a result of several products produced by *B. velezensis* with antibacterial effects, such as bacteriocin produced by *B. velezensis* strain BUU004 isolated from shrimp pond sediment [97, 98].

AST and ALT enzymes reflect the health status of the shrimp hepatopancreas [99]. Dietary *B. velezensis* decreased the activities of these two enzymes in *L. vannamei*, indicating improved function of the hepatopancreas. The results obtained were identical to those reported by Chen et al. [25] and Yang et al. [14].

Crustaceans’ immune systems rely primarily on phosphorylation and dephosphorylation, both of which require AKP and ACP [100]. Furthermore, AKP is a regulatory enzyme involved in metabolism and phagolysis, whereas ACP is a key component of lysozyme enzymes that destroy pathogens in invertebrates [101, 102]. In this study, all groups fed the *B. velezensis* diet had higher AKP and ACP levels than the control group. Chen et al. [25] reported similar results for *L. vannamei* fed a *B. velezensis-*rich diet. Amoah et al. [17] also found an increase in ACP in *L. vannamei*-fed diets enriched with *B. coagulans*.

In the current study, all groups of *L. vannamei* fed a *B. velezensis-*enriched diet showed significant decreases in triglycerides, cholesterol, high-density lipoprotein, and low-density lipoprotein compared with the control group, suggesting that *B. velezensis* may play a vital role in regulating lipid metabolism. Lipid metabolism is positively correlated with blood triglyceride levels [103]. *B. velezensis* YFI-E109 in diets enhanced lipid utilization and reduced lipid deposition in fish, resulting in decreased triglyceride levels in hybrid yellow catfish [104]. In the same regard, Amoah et al. [17] reported lower triglyceride levels in *L. vannamei*-fed *B. coagulans*-enriched diets. Lee et al. [60] reported that *Bacillus* strains can synthesize vitamins and thus lower cholesterol levels.

The hepatopancreas is a vital organ in shrimp that regulates several functions of the digestive system, including steroid hormone production, digestive enzyme secretion, carbohydrate and fat metabolism, and nutrient absorption, distribution, and storage. In addition, the hepatopancreas is the main detoxification organ of shrimp [34, 105, 106].

In this study, groups of *L. vannamei* fed *B. velezensis*-enriched diets showed higher numbers of B cells (secreting cells; the main producer of digestive enzymes) and R cells (absorption, lipid and glycogen storage cells), which are involved in the digestion, absorption, and storage of nutrients, which in turn indicated the improvement in growth performance in these groups compared with the control group, which had an increase in undifferentiated embryonic cells (E). The above result is consistent with those of Chen et al. [19], in addition to García-Bernal et al. [26], who reported an increase in B cells in *L. vannamei* fed with Streptomyces probiotics relative to the control group.

## Conclusion

In summary, the current study indicated that *Bacillus velezensis* is a promising probiotic that can be safely added to the diet of *Litopenaeus vannamei* with 1 × 10^9^ CFU/g. Its application had a positive influence on the health status, survival rate, nutritional value, and immunity of *L. vannamei*.

## Data Availability

The data sets used in the present study are accessible on reasonable request from the corresponding author.
